# A double machine learning model for measuring the impact of the Made in China 2025 strategy on green economic growth

**DOI:** 10.1038/s41598-024-62916-0

**Published:** 2024-05-26

**Authors:** Jie Yuan, Shucheng Liu

**Affiliations:** 1 School of Public Finance and Taxation, Zhejiang University of Finance and Economics, Hangzhou, 310018 China; 2 School of Economics, Xiamen University, Xiamen, 361005 China

**Keywords:** Made in China 2025, Industrial policy, Green economic growth, Double machine learning, Causal inference, Environmental economics, Environmental impact, Sustainability

## Abstract

The transformation and upgrading of China’s manufacturing industry is supported by smart and green manufacturing, which have great potential to empower the nation’s green development. This study examines the impact of the Made in China 2025 industrial policy on urban green economic growth. This study applies the super-slacks-based measure model to measure cities’ green economic growth, using the double machine learning model, which overcomes the limitations of the linear setting of traditional causal inference models and maintains estimation accuracy under high-dimensional control variables, to conduct an empirical analysis based on panel data of 281 Chinese cities from 2006 to 2021. The results reveal that the Made in China 2025 strategy significantly drives urban green economic growth, and this finding holds after a series of robustness tests. A mechanism analysis indicates that the Made in China 2025 strategy promotes green economic growth through green technology progress, optimizing energy consumption structure, upgrading industrial structure, and strengthening environmental supervision. In addition, the policy has a stronger driving effect for cities with high manufacturing concentration, industrial intelligence, and digital finance development. This study provides valuable theoretical insights and policy implications for government planning to promote high-quality development through industrial policy.

## Introduction

Since China’s reform and opening up, the nation’s economy has experienced rapid growth for more than 40 years. According to the National Bureau of Statistics, China’s per capita GDP has grown from 385 yuan in 1978 to 85,698 yuan in 2022, with an average annual growth rate of 13.2%. However, obtaining this growth miracle has come at considerable social and environmental costs^1^. Current pollution prevention and control systems have not yet fundamentally alleviated the structural and root causes, impairing China’s economic progress toward high-quality development^2^. The report of the 20th National Congress of the Communist Party of China proposed that the future will be focused on promoting the formation of green modes of production and lifestyles and advancing the harmonious coexistence of human beings and nature. This indicates that transforming the mode of economic development is now the focus of the government’s attention, calling for advancing the practices of green growth aimed at energy conservation, emissions reduction, and sustainability while continuously increasing economic output^3^. As a result, identifying approaches to balance economic growth and green environmental protection in the development process and realize green economic growth has become an arduous challenge and a crucially significant concern for China’s high-quality economic development.

An intrinsic driver of urban economic growth, manufacturing is also the most energy-intensive and pollution-emitting industry, and greatly constrains urban green development^4^. China’s manufacturing industry urgently needs to advance the formation of a resource-saving and environmentally friendly industrial structure and manufacturing system through transformation and upgrading to support for green economic growth^5^. As an incentive-based industrial policy that emphasizes an innovation-driven and eco-civilized development path through the development and implementation of an intelligent and green manufacturing system, Made in China 2025 is a significant initiative for promoting the manufacturing industry’s transformation and upgrading, providing solid economic support for green economic growth^6^. To promote the effective implementation of this industrial policy, fully mobilize localities to explore new modes and paths of manufacturing development, and strengthen the urban manufacturing industry’s influential demonstration role in advancing the green transition, the Ministry of Industry and Information Technology of China successively launched 30 Made in China 2025 pilot cities (city clusters) in 2016 and 2017. The Pilot Demonstration Work Program for “Made in China 2025” Cities specified that significant results should be achieved within three to 5 years. After several years of implementation, has the Made in China 2025 pilot policy promoted green economic growth? What are the policy’s mechanisms of action? Are there differences in green economic growth effects in pilot cities based on various urban development characteristics? This study’s theoretical interpretation and empirical examination of the above questions can add to the growing body of related research and provide valuable insights for cities to comprehensively promote the transformation and upgrading of manufacturing industry to advance China’s high-quality development.

This study constructs an analytical framework at the theoretical level to analyze the impact of the Made in China 2025 strategy on urban green economic growth, and uses the double machine learning (ML) model to test its green economic growth effect. The contributions of this study are as follows. First, focusing on the field of urban green development, the study incorporates variables representing the potential economic and environmental effects of the Made in China 2025 policy into a unified framework to systematically examine the impact of the Made in China 2025 pilot policy on the urban green economic growth, providing a novel perspective for assessing the effects of industrial policies. Second, we investigate potential transmission mechanisms of the Made in China 2025 strategy affecting green economic growth from the perspectives of green technology advancement, energy consumption structure optimization, industrial structure upgrading, and environmental supervision strengthening, establishing a useful supplement for related research. Third, leveraging the advantage of ML algorithms in high-dimensional and nonparametric prediction, we apply a double ML model assess the policy effects of the Made in China 2025 strategy to avoid the “curse of dimensionality” and the inherent biases of traditional econometric models, and improve the credibility of our research conclusions.

The remainder of this paper is structured as follows. Section “Literature review” presents a literature review. Section “Policy background and theoretical analysis” details our theoretical analysis and research hypotheses. Section “Empirical strategy” introduces the model setting and variables selection for the study. Section “Empirical result” describes the findings of empirical testing and analyzes the results. Section “Conclusion and policy recommendation” summarizes our conclusions and associated policy implications.

## Literature review

### Measurement and influencing factors of green economic growth

The Green Economy Report, which was published by the United Nations Environment Program in 2011, defined green economy development as facilitating more efficient use of natural resources and sustainable growth than traditional economic models, with a more active role in promoting combined economic development and environmental protection. The Organization for Economic Co-operation and Development defined green economic growth as promoting economic growth while ensuring that natural assets continue to provide environmental resources and services; a concept that is shared by a large number of institutions and scholars^7–9^. A considerable amount of research has assessed green economic growth, primarily using three approaches. First, single-factor indicators, such as sulfur dioxide emissions, carbon dioxide emissions intensity, and other quantified forms; however, this approach neglects the substitution of input factors such as capital and labor for the energy factor, which has certain limitations^5,10^. Second, studies have been based on neoclassical economic growth theory, incorporating factors of capital, technology, energy, and the environment, and constructing a green Solow model to measure green total factor productivity (GTFP)^11,12^. Third, based on neoclassical economic growth theory, some studies have simultaneously considered desirable and undesirable output, applying Shepard’s distance function, the directional distance function, and data envelopment analysis to measure GTFP^13–15^.

Economic growth is an extremely complex process, and green economic growth is also subject to a combination of multiple complex factors. Scholars have explored the influence mechanisms of green economic growth from perspectives of resource endowment^16^, technological innovation^17^, industrial structure^18^, human capital^19^, financial support^20^, government regulation^21^, and globalization^22^. In the field of policy effect assessment, previous studies have confirmed the green development effects of pilot policies such as innovative cities^23^, Broadband China^24^, smart cities^25^, and low-carbon cities^26^. However, few studies have focused on the impact of Made in China 2025 strategy on urban green economic growth and identified its underlying mechanisms.

### The impact of Made in China 2025 strategy

Since the industrial policy of Made in China 2025 was proposed, scholars have predominantly focused on exploring its economic effects on technological innovation^27^, digital transformation^28^, and total factor productivity (TFP)^29^, while the potential environmental effects have been neglected. Chen et al. (2024)^30^ found that Made in China 2025 promotes firm innovation through tax incentives, public subsidies, convenient financing, academic collaboration and talent incentives. Xu (2022)^31^ point out that Made in China 2025 policy has the potential to substantially improve the green innovation of manufacturing enterprises, which can boost the green transformation and upgrading of China’s manufacturing industry. Li et al. (2024)^32^ empirically investigates the positive effect of Made in China 2025 strategy on digital transformation and exploratory innovation in advanced manufacturing firms. Moreover, Liu and Liu (2023)^33^ take “Made in China 2025” as an exogenous shock and find that the pilot policy has a positive impact on the high-quality development of enterprises and capital markets. Unfortunately, scholars have only discussed the impact of Made in China 2025 strategy on green development and environmental protection from a theoretical perspective and lack empirical analysis. Li (2018)^27^ has compared Germany’s “Industry 4.0” and China’s “Made in China 2025”, and point out that “Made in China 2025” has clear goals, measures and sector focus. Its guiding principles are to enhance industrial capability through innovation-driven manufacturing, optimize the structure of Chinese industry, emphasize quality over quantity, train and attract talent, and achieve green manufacturing and environment. Therefore, it is necessary to systematically explore the impact and mechanism of Made in China 2025 strategy on urban green economic growth from both theoretical and empirical perspectives.

### Causal inference based on double ML

The majority of previous studies have used traditional causal inference models to assess policy effects; however, some limitations are inherent to the application of these models. For example, the parallel trend test of the difference-in-differences model has stringent requirements on appropriate sample data; the synthetic control method can construct a virtual control group that conforms to the parallel trend, but it requires that the treatment group does not have the extreme value characteristics, and it is only applicable to “one-to-many” circumstances; and the propensity score matching (PSM) method involves a considerable amount of subjectivity in selecting matching variables. To compensate for the shortcomings of traditional models, scholars have started to explore the application of ML in the field of causal inference^34–36^, and double ML is a typical representative.

Double ML was formalized in 2018^34^, and the relevant research falls into two main categories. The first strand of literature applies double ML to assess causality concerning economic phenomena. Yang et al. (2020)^37^ applied double ML using a gradient boosting algorithm to explore the average treatment effect of top-ranked audit firms, verifying its robustness compared with the PSM method. Zhang et al. (2022)^38^ used double ML to quantify the impact of nighttime subway services on the nighttime economy, house prices, traffic accidents, and crime following the introduction of nighttime subway services in London in 2016. Farbmacher et al. (2022)^39^ combined double ML with mediating effects analysis to assess the causal relationship between health insurance coverage and youth wellness and examine the indirect mechanisms of regular medical checkups, based on a national longitudinal health survey of youth conducted by the US Bureau of Labor Statistics. The second strand of literature has innovated methodological theory based on double ML. Chiang et al. (2022)^40^ proposed an improved multidirectional cross-fitting double ML method, obtaining regression results for high-dimensional parameters while estimating robust standard errors for dual clustering, which can effectively adapt to multidirectional clustered sampled data and improve the validity of estimation results. Bodory et al. (2022)^41^ combined dynamic analysis with double ML to measure the causal effects of multiple treatment variables over time, using weighted estimation to assess the dynamic treatment effects of specific subsamples, which enriched the dynamic quantitative extension of double ML.

In summary, previous research has conducted some useful investigations regarding the impact of socioeconomic policies on green development, but limited studies have explored the relationship between the Made in China 2025 strategy and green economic growth. This study takes 281 Chinese cities as the research object, and applies the super-slacks-based measure (SBM) model to quantify Chinese cities’ green economic growth from 2006 to 2021. Based on a quasi-natural experiment of Made in China 2025 pilot policy implementation, we use the double ML model to test the impact and transmission mechanisms of the policy on urban green economic growth. We also conduct a heterogeneity analysis of cities based on different levels of manufacturing agglomeration, industrial intelligence, and digital finance. This study applies a novel approach and provides practical insights for research in the field of industrial policy assessment.

## Policy background and theoretical analysis

### Policy background

The Made in China 2025 strategy aims to encourage and support local exploration of new paths and models for the transformation and upgrading of the manufacturing industry, and to drive the improvement of manufacturing quality and efficiency in other regions through demonstration effects. According to the Notice of Creating “Made in China 2025” National Demonstration Zones issued by the State Council, municipalities directly under the central government, sub-provincial cities, and prefecture-level cities can apply for the creation of demonstration zones. Cities with proximity and high industrial correlation can jointly apply for urban agglomeration demonstration zones. The Notice clarifies the goals and requirements for creating demonstration zones in areas such as green manufacturing, clean production, and environmental protection. In 2016, Ningbo became the first Made in China 2025 pilot city, and a total of 12 cities and 4 city clusters were included in the list of Made in China 2025 national demonstration zones. In 2018, the State Council issued the Evaluation Guidelines for “Made in China 2025” National Demonstration Zone, which further clarified the evaluation process and indicator system of the demonstration zone. Seven primary indicators and 29 secondary indicators were formulated, including innovation driven, quality first, green development, structural optimization, talent oriented, organizational implementation, and coordinated development of urban agglomerations. This indicator system can evaluate the creation process and overall effectiveness of pilot cities (city clusters), which is beneficial for the promotion of successful experiences and models in demonstration areas.

Advancing green urban development is a complex systematic project that requires structural adjustment and technological and institutional changes in the socioeconomic system^42^. The Made in China 2025 strategy emphasizes the development and application of smart and green manufacturing systems, which can unblock technological bottlenecks in the manufacturing sector in terms of industrial production, energy consumption, and waste emissions, and empower cities to operate in a green manner. In addition, the Made in China 2025 policy established requirements for promoting technological innovation to advance energy saving and environmental protection, improving the rate of green energy use, transforming traditional industries, and strengthening environmental supervision. For pilot cities, green economy development requires the support of a full range of positive factors. Therefore, this study analyzes the mechanisms by which the Made in China 2025 strategy affects urban green economic growth from the four paths of green technology advancement, energy consumption structure optimization, industrial structure upgrading, and environmental supervision strengthening.

### Theoretical analysis and research hypotheses

As noted, the Made in China 2025 strategy emphasizes strengthening the development and application of energy-saving and environmental protection technologies to advance cleaner production. Pilot cities are expected to prioritize the driving role of green innovation, promote clustering carriers and innovation platforms for high-tech enterprises, and guide the progress of enterprises’ implementation of green technology. Specifically, pilot cities are encouraged to optimize the innovation environment by increasing scientific and technological investment and financial subsidies in key areas such as smart manufacturing and high-end equipment and strengthening intellectual property protection to incentivize enterprises to conduct green research and development (R&D) activities. These activities subsequently promote the development of green innovation technologies and industrial transformation^43^. Furthermore, since quality human resources are a core aspect of science and technology innovation^44^, pilot cities prioritize the cultivation and attraction of talent to establish a stable human capital guarantee for enterprises’ ongoing green technology innovation, transform and upgrade the manufacturing industry, and advance green urban development. Green technology advances also contribute to urban green economic growth. First, green technology facilitates enterprises’ adoption of improved production equipment and innovation in green production technology, accelerating the change of production mode and driving the transformation from traditional crude production to a green and intensive approach^45^, promoting green urban development. Second, green technology advancement accelerates green innovations such as clean processes, pollution control technologies, and green equipment, and facilitates the effective supply of green products, taking full advantage of the benefits of green innovations^46^ and forming a green economic development model to achieve urban green economic growth.

The Made in China 2025 pilot policy endeavors to continuously increase the rate of green and low-carbon energy use and reduce energy consumption. Under target constraints of energy saving and carbon control, pilot cities will accelerate the cultivation of high-tech industries in green environmental protection and high-end equipment manufacturing with advantages of sustainability and low resource inputs^47^ to improve the energy consumption structure. Pilot cities also advance new energy sector development by promoting clean energy projects, subsidizing new energy consumption, and supporting green infrastructure construction and other policy measures^48^ to optimize the energy consumption structure. Energy consumption structure optimization can have a profound impact on green economy development. Optimization means that available energy tends to be cleaner, which can reduce the manufacturing industry’s dependence on traditional fossil energy and raise the proportion of clean energy^49^, ultimately promoting green urban development. Pilot cities also provide financial subsidies for new energy technology R&D, which promotes the innovation and application of new technologies, energy-saving equipment, efficient resource use, and energy-saving diagnostics, which allow enterprises to save energy and reduce consumption and improve energy use efficiency and TFP^50^, advancing the growth of urban green economy.

At its core, the Made in China 2025 strategy promotes the transformation and upgrading of the manufacturing sector. Pilot cities guide and develop technology-intensive high-tech industries, adjust the proportion of traditional heavy industry, and improve the urban industrial structure. Pilot cities also implement the closure, merger, and transformation of pollution-intensive industries; guide the fission of professional advantages of manufacturing enterprises^51^; and expand the establishment and development of service-oriented manufacturing and productive service industries to promote the evolution of the industrial structure toward rationalization and high-quality development^52^. Upgrading the industrial structure can also contribute to urban green economic growth. First, industrial structure upgrading promotes the transition from labor- and capital-intensive industries to knowledge- and technology-intensive industries, which optimizes the industrial distribution patterns of energy consumption and pollutant emissions and promotes the transformation of economic growth dynamics and pollutant emissions control, providing a new impetus for cities’ sustainable development^53^. Second, changes in industrial structure and scale can have a profound impact on the type and quantity of pollutant emissions. By introducing high-tech industries, service-oriented manufacturing, and production-oriented service industries, pilot cities can promote the transformation of pollution-intensive industries, promoting the adjustment and optimization of industrial structure and scale^54^ to achieve the purpose of driving green urban development.

The Made in China 2025 strategy proposes strengthening green supervision and conducting green evaluations, establishing green development goals for the manufacturing sector in terms of emissions and consumption reduction and water conservation. This requires pilot cities to implement stringent environmental regulatory policies, such as higher energy efficiency and emissions reduction targets and sewage taxes and charges, strict penalties for excess emissions, and project review criteria^55^, which consolidates the effectiveness of green development. Under the framework of environmental authoritarianism, strengthening environmental supervision is a key measure for achieving pollution control and improving environmental quality^56^. Therefore, environmental regulatory enhancement can help cities achieve green development goals. First, according to the Porter hypothesis^57^, strong environmental regulatory policies encourage firms to internalize the external costs of environmental supervision, stimulate technological innovation, and accelerate R&D and application of green technologies. This response helps enterprises improve input–output efficiency, achieve synergy between increasing production and emissions reduction, partially or completely offset the “environmental compliance cost” from environmental supervision, and realize the innovation compensation effect^58^. Second, strict environmental regulations can effectively mitigate the complicity of local governments and enterprises in focusing on economic growth while neglecting environmental protection^59^, urging local governments to constrain enterprises’ emissions, which compels enterprises to conduct technological innovation and pursue low-carbon transformation, promoting urban green economic growth.

**Figure 1:**
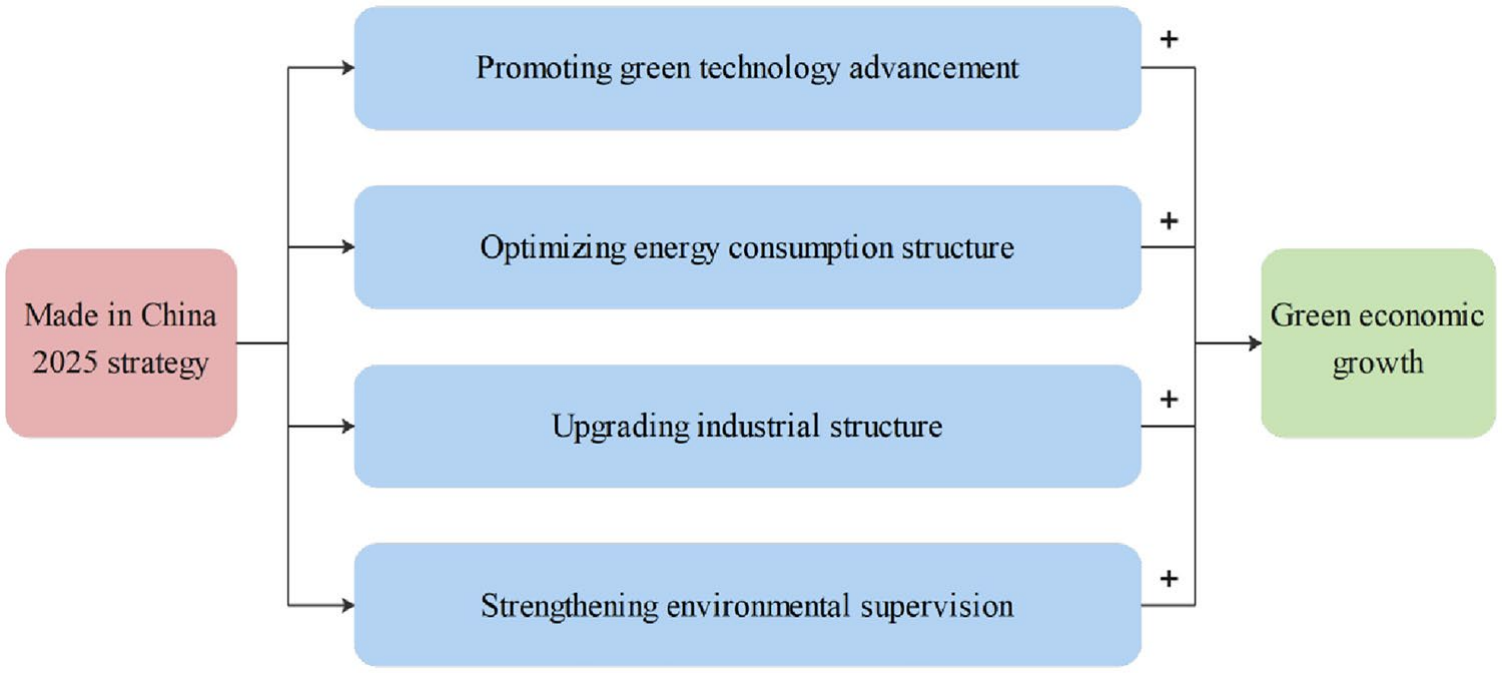
Mechanism analysis of Made in China 2025 strategy and green economic growth.

Based on the above analysis, we propose the mechanisms that promote green economic growth through Made in China 2025 strategy, as shown in Fig. 1. The proposed research hypotheses are as follows:

#### Hypothesis 1

The Made in China 2025 strategy promotes urban green economic growth.

#### Hypothesis 2

The Made in China 2025 strategy drives urban green economic growth through four channels: promoting green technology advancement, optimizing energy consumption structure, upgrading industrial structure, and strengthening environmental supervision.

## Empirical strategy

### Double ML model

Compared with traditional causal inference models, double ML has unique advantages in variable selection and model estimation, and is also more applicable to the research problem of this study. Green economic growth is a comprehensive indicator of transformative urban growth that is influenced by many socioeconomic factors. To ensure the accuracy of our policy effects estimation, the interference of other factors on urban green economic growth must be controlled as much as possible; however, when introducing high-dimensional control variables, traditional regression models may face the “curse of dimensionality” and multicollinearity, rendering the accuracy of the estimates questionable. Double ML uses ML and regularization algorithms to automatically filter the preselected set of high-dimensional control variables to obtain an effective set of control variables with higher prediction accuracy. This approach avoids the “curse of dimensionality” caused by redundant control variables and mitigates the estimation bias caused by the limited number of primary control variables^39^. Furthermore, nonlinear relationships between variables are the norm in the evolution of economic transition, and ordinary linear regression may suffer from model-setting bias producing estimates that lack robustness. Double ML effectively overcomes the problem of model misspecification by virtue of the advantages of ML algorithms in handling nonlinear data^37^. In addition, based on the idea of instrumental variable functions, two-stage predictive residual regression, and sample split fitting, double ML mitigates the “regularity bias” in ML estimation and ensures unbiased estimates of the treatment coefficients in small samples^60^.

Based on the analysis above, this study uses the double ML model to assess the policy effects of the Made in China 2025 strategy. The partial linear double ML model is constructed as follows:

1 $$ Y_{it} = \theta_{0} Policy_{it} + g(X_{it} ) + U_{it} $$

2 $$ E(U_{it} \left| {Policy_{it} ,X_{it} } \right.) = 0 $$

where *i* denotes the city, *t* denotes the year, and *Y*_*it*_ represents green economic growth. *Policy*_*it*_ represents the policy variable of Made in China 2025, which is set as 1 if the pilot is implemented and 0 otherwise. *θ*_0_ is the treatment coefficient that is the focus of this study. *X*_*it*_ denotes the set of high-dimensional control variables, and the ML algorithm is used to estimate the specific functional form $\hat{g}(X_{it} )$. *U*_*it*_ denotes the error term with a conditional mean of zero.

Direct estimation of Eqs. (1) and (2) yields the following estimate of the treatment coefficient:

3 $$ \hat{\theta }_{0} = \left( {\frac{1}{n}\sum\nolimits_{i \in I,t \in T} {Policy_{it}^{2} } } \right)^{ - 1} \frac{1}{n}\sum\nolimits_{i \in I,t \in T} {Policy_{it} (Y_{it} - \hat{g}(X_{it} ))} $$

where *n* denotes the sample size.

Notably, the double ML model uses a regularization algorithm to estimate the specific functional form $\hat{g}(X_{it} )$, which prevents the variance of the estimate from being too large, but inevitably introduces a “regularity bias,” resulting in a biased estimate. To speed up the convergence of the $\hat{g}(X_{it} )$ directions so that the estimates of the treatment coefficients satisfy unbiasedness with small samples, the following auxiliary regression is constructed:

4 $$ Policy_{it} = m(X_{it} ) + V_{it} $$

5 $$ E(V_{it} \left| {X_{it} } \right.) = 0 $$

where $m(X_{it} )$ is the regression function of the treatment variable on the high-dimensional control variable, using ML algorithms to estimate the specific functional form $\hat{m}(X_{it} )$. *V*_*it*_ is the error term with a conditional mean of zero.

The specific operation process follows three stages. First, we use the ML algorithm to estimate the auxiliary regression $\hat{m}(X_{it} )$ and take its residuals $\hat{V}_{it} = Policy_{it} - \hat{m}(X_{it} )$. Second, we use the ML algorithm to estimate $\hat{g}(X_{it} )$ and change the form of the main regression $Y_{it} - \hat{g}(X_{it} ) = \theta_{0} Policy_{it} + U_{it}$. Finally, we regress $\hat{V}_{it}$ as an instrumental variable for *Policy*_*it*_, obtaining unbiased estimates of the treatment coefficients as follows:

6 $$ \tilde{\theta }_{0} = \left( {\frac{1}{n}\sum\nolimits_{i \in I,t \in T} {\hat{V}_{it} Policy_{it}^{2} } } \right)^{ - 1} \frac{1}{n}\sum\nolimits_{i \in I,t \in T} {\hat{V}_{it} (Y_{it} - \hat{g}(X_{it} ))} $$

### Variable selection

#### Green economic growth

We apply the super-SBM model to measure urban green economic growth. The super-SBM model is compatible with radial and nonradial characteristics, which avoids inflated results due to ignoring slack variables and deflated results due to ignoring the linear relationships between elements, and can truly reflect relative efficiency^61^. The SBM model reflects the nature of green economic growth more accurately compared with other models, and has been widely adopted by scholars^62^. The expression of the super-SBM model considering undesirable output is as follows:

7 $$ \begin{array}{*{20}c} {\begin{array}{*{20}c} {\begin{array}{*{20}c} {\min \rho_{SE} = \frac{{1 + \frac{1}{m}\sum\limits_{i = 1}^{m} {s_{i}^{ - } /x_{ik} } }}{{1 - \frac{1}{{s_{1} + s_{2} }}\left( {\sum\limits_{r = 1}^{{s_{1} }} {s_{r}^{ + } /y_{rk} } + \sum\limits_{t = 1}^{{s_{2} }} {s_{t}^{z - } /z_{tk} } } \right)}}} \\ {s.t.} \\ \end{array} } \\ {\sum\limits_{j = 1,j \ne k}^{n} {x_{ij} \gamma_{j} - s_{i}^{ - } \le x_{ik} } } \\ \begin{gathered} \sum\limits_{j = 1,j \ne k}^{n} {y_{rj} \gamma_{j} + s_{r}^{ + } \ge y_{rk} } \hfill \\ \sum\limits_{j = 1,j \ne k}^{n} {z_{rj} \gamma_{j} + s_{t}^{z - } \le z_{rk} } \hfill \\ \end{gathered} \\ \end{array} } \\ {\gamma ,s^{ - } ,s^{ + } ,s^{z - } \ge 0} \\ {i = 1,2, \cdots ,q;\;j = 1,2, \cdots ,n(j \ne k)} \\ \end{array} $$

where *x* is the input variable; *y* and *z* are the desirable and undesirable output variables, respectively; *m* denotes the number of input indicators; *s*_1_ and *s*_2_ represent the respective number of indicators for desirable and undesirable outputs; *k* denotes the period of production; *i*, *r*, and *t* are the decision units for the inputs, desirable outputs, and undesirable outputs, respectively; $s^{ - }$, $s^{ + }$, and $s^{z - }$ are the respective slack variables for the inputs, desirable outputs, and undesirable outputs; and *γ* is a vector of weights. A larger $\rho_{SE}$ value indicates greater efficiency. If $\rho_{SE}$ = 1, the decision unit is effective; if $\rho_{SE}$ < 1, the decision unit is relatively ineffective, indicating a loss of efficiency.

Referencing Sarkodie et al. (2023)^63^, the evaluation index system of green economic growth is constructed as shown in Table 1.

**Table 1:** Indicator system for green economic growth.

Variables	Indicators	Definitions
Input	Labor	Number of employees at the end of the year
	Capital stock	Estimated using the perpetual inventory method at constant prices in 2005
	Energy	Per capita consumption of electricity
Desirable output	GDP	Real GDP at constant prices in 2005
Undesirable output	Pollution index	Based on industrial wastewater, smoke and dust, sulfur dioxide emissions and carbon emissions, the entropy method is used to measure the comprehensive pollution index

#### Made in China 2025 pilot policy

The list of Made in China 2025 pilot cities (city clusters) published by the Ministry of Industry and Information Technology of China in 2016 and 2017 is matched with the city-level data to obtain 30 treatment group cities and 251 control group cities. The policy dummy variable of Made in China 2025 is constructed by combining the implementation time of the pilot policies.

#### Mediating variables

This study also examines the transmission mechanism of the Made in China 2025 strategy affecting urban green economic growth from four perspectives, including green technology advancement, energy consumption structure optimization, industrial structure upgrading, and strengthening of environmental supervision. (1) The number of green patent applications is adopted to reflect green technology advancement. (2) Energy consumption structure is quantified using the share of urban domestic electricity consumption in total energy consumption. (3) The industrial structure upgrading index is calculated using the formula $\sum\nolimits_{i = 1}^{3} {i \times (GDP_{i} /GDP)}$, where *GDP*_*i*_ denotes the added value of primary, secondary, or tertiary industries. (4) The frequency of words related to the environment in government work reports is the proxy for measuring the intensity of environmental supervision^64^.

#### Control variables

Double ML can effectively accommodate the case of high-dimensional control variables using regularization algorithms. To control for the effect of other urban characteristics on green economic growth, this study introduces the following 10 control variables. We measure education investment by the ratio of education expenditure to GDP. Technology investment is the ratio of technology expenditure to GDP. The study measures urbanization using the share of urban built-up land in the urban area. Internet penetration is the number of internet users as a share of the total population at the end of the year. We measure resident consumption by the total retail sales of consumer goods per capita. The unemployment rate is the ratio of the number of registered unemployed in urban areas at the end of the year to the total population at the end of the year. Financial scale is the ratio of the balance of deposits and loans of financial institutions at the end of the year to the GDP. Human capital is the natural logarithm of the number of students enrolled in elementary school, general secondary schools, and general tertiary institutions per 10,000 persons. Transportation infrastructure is the natural logarithm of road and rail freight traffic. Finally, openness to the outside world is reflected by the ratio of actual foreign investment to GDP. Quadratic terms for the control variables are also included in the regression analysis to improve the accuracy of the model’s fit. We introduce city and time fixed effects as individual and year dummy variables to avoid missing information on city and time dimensions.

### Data sources

This study uses 281 Chinese cities spanning from 2006 to 2021 as the research sample. Data sources include the China City Statistical Yearbook, the China Economic and Social Development Statistics Database, and the EPS Global Statistics Database. We used the average annual growth rate method to fill the gaps for the minimal missing data. To remove the effects of price changes, all data measured in monetary units are deflated using the consumer price index for each province for the 2005 base period. The descriptive statistics of the data are presented in Table 2.

**Table 2:** Descriptive statistics of variables.

Variable	Abb	N	Mean	Std. dev	Min	Max
Green economic growth	Geco	4496	0.804	0.318	0.183	3.316
Made in China 2025 pilot policy	Policy	4496	0.028	0.166	0.000	1.000
Green technological advancement	Gtech	4496	2.028	1.233	0.881	7.778
Energy consumption structure	Energy	4496	0.253	0.304	0.227	1.000
Industrial structure	Indus	4496	2.188	0.772	0.881	3.258
Environmental supervision	Superv	4496	3.114	1.363	1.277	20.708
Education investment	Edu	4496	0.035	0.019	0.008	0.185
Technology investment	Sci	4496	0.003	0.003	0.000	0.063
Urbanization	Urb	4496	5.738	0.922	1.620	7.882
Internet penetration	Int	4496	0.779	0.288	0.087	2.243
Resident consumption	Cons	4496	0.212	0.184	0.003	3.664
Unemployment rate	Unem	4496	0.383	0.110	0.000	1.013
Financial scale	Fina	4496	0.964	0.537	0.176	5.154
Human capital	Hum	4496	0.202	0.135	0.044	2.527
Transportation infrastructure	Tran	4496	0.009	0.123	0.000	6.509
Openness to the outside world	Open	4496	2.381	1.248	0.588	21.301

## Empirical result

### Baseline results

The sample split ratio of the double ML model is set to 1:4, and we use the Lasso algorithm to predict and solve the main and auxiliary regressions, presenting the results in Table 3. Column (1) does not control for fixed effects or control variables, column (2) introduces city and time fixed effects, and columns (3) and (4) add control variables to columns (1) and (2), respectively. The regressions in columns (1) and (2) are highly significant, regardless of whether city and time fixed effects are controlled. Column (4) controls for city fixed effects, time fixed effects, and the primary term of the control variable over the full sample interval, revealing that the regression coefficient of the Made in China 2025 pilot policy on green economic growth is positive and significant at the 1% level, confirming that the Made in China 2025 strategy significantly promotes urban green economic growth. Column (5) further incorporates the quadratic terms of the control variables and the regression coefficients remain significantly positive with little change in values. Therefore, Hypothesis 1 is verified.

**Table 3:** Benchmark regression results.

Variables	(1)	(2)	(3)	(4)	(5)
Policy	0.3881*** (0.0519)	0.1862*** (0.0370)	0.1763*** (0.0328)	0.1235*** (0.0356)	0.1194*** (0.0347)
Control variable linear term	No	No	Yes	Yes	Yes
Control variable quadratic term	No	No	No	No	Yes
City FE	No	Yes	No	Yes	Yes
Year FE	No	Yes	No	Yes	Yes
N	4496	4496	4496	4496	4496

***, **, and * indicate statistical significance at 1%, 5%, and 10% levels, respectively. Robust standard errors are in parentheses.

### Parallel trend test

**Figure 2:**
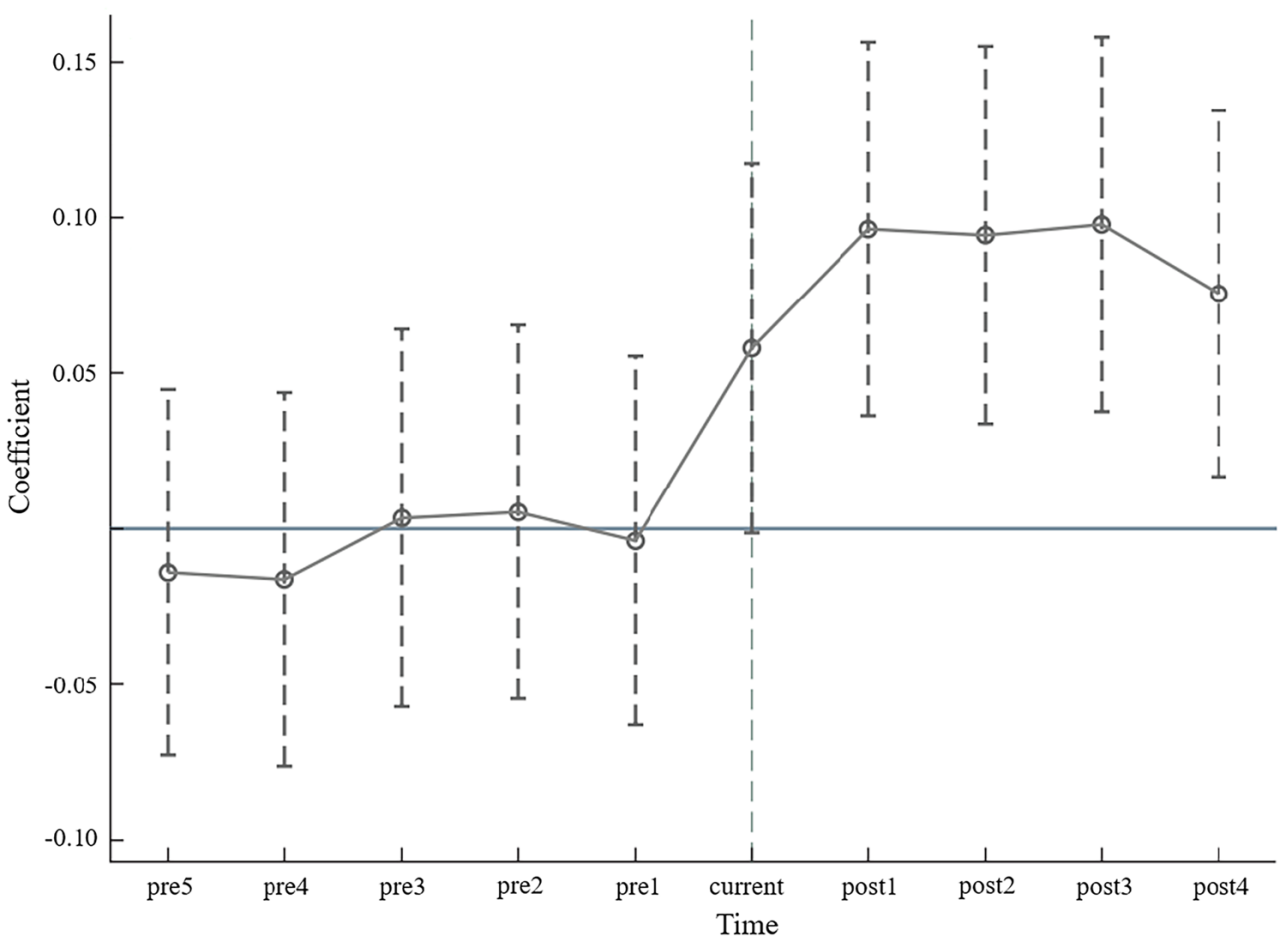
Parallel trend test.

The prerequisite for the establishment of policy evaluation is that the development status of cities before the pilot policy is introduced is similar. Referring to Liu et al. (2022)^29^, we adopt a parallel trend test to verify the effectiveness of Made in China 2025 pilot policy. Figure 2 shows the result of parallel trend test. None of the coefficient estimates before the Made in China 2025 pilot policy are significant, indicating no significant difference between the level of green economic growth in pilot and nonpilot cities before implementing the policy, which passes the parallel trend test. The coefficient estimates for all periods after the policy implementation are significantly positive, indicating that the Made in China 2025 pilot policy can promote urban green economic growth.

### Robustness tests

#### Replace explained variable

Referencing Oh and Heshmati (2010)^65^ and Tone and Tsutsui (2010)^66^, we use the Malmquist–Luenberger index under global production technology conditions (GML) and an epsilon-based measure (EBM) model to recalculate urban green economic growth. The estimation results in columns (1) and (2) of Table 4 show that the estimated coefficients of the Made in China 2025 pilot policy remain significantly positive after replacing the explanatory variables, validating the robustness of the baseline findings.

**Table 4:** Robustness test results 1.

Variables	(1)	(2)	(3)	(4)
	GML	EBM	Excluding special cities	2013–2020
Policy	0.0557*** (0.0081)	0.0892*** (0.0193)	0.1063*** (0.0246)	0.0705*** (0.0256)
Control variable linear term	Yes	Yes	Yes	Yes
Control variable quadratic term	Yes	Yes	Yes	Yes
City FE	Yes	Yes	Yes	Yes
Year FE	Yes	Yes	Yes	Yes
N	4496	4496	3952	2248

***, **, and * indicates statistical significance at 1%, 5%, and 10% levels, respectively. Robust standard errors are in parentheses.

#### Adjusting the research sample

Considering the large gaps in the manufacturing development base between different regions in China, using all cities in the regression analysis may lead to biased estimation^67^. Therefore, we exclude cities in seven provinces with a poor manufacturing development base (Gansu, Qinghai, Ningxia, Xinjiang, Tibet, Yunnan, and Guizhou) and four municipalities with a better development base (Beijing, Tianjin, Shanghai, and Chongqing). The other city samples are retained to rerun the regression analysis, and the results are presented in column (3) of Table 4. The first batch of pilot cities of the Made in China 2025 strategy was released in 2016, and the second batch of pilot cities was released in 2017. To exclude the effect of point-in-time samples that are far from the time of policy promulgation, the regression is also rerun by restricting the study interval to the three years before and after the promulgation of the policy (2013–2020), and the results are presented in column (4) of Table 4. The coefficients of the Made in China 2025 pilot policy effect on urban green economic growth decrease after adjusting for the city sample and the time interval, but remain significantly positive at the 1% level. This, once again, verifies the robustness of the benchmark regression results.

#### Eliminating the impact of potential policies

During the same period of the Made in China 2025 strategy implementation, urban green economy growth may be affected by other relevant policies. To ensure the accuracy of the policy effect estimates, four representative policy categories overlapping with the sample period, including smart cities, low-carbon cities, Broadband China, and innovative cities, were collected and organized. Referencing Zhang and Fan (2023)^25^, dummy variables for these policies are included in the benchmark regression model and the results are presented in Table 5. The estimated coefficient of the Made in China 2025 pilot policy decreases after controlling for the effects of related policies, but remains significantly positive at the 1% level. This suggests that the positive impact of the Made in China 2025 strategy on urban green economic growth, although overestimated, does not affect the validity of the study’s findings.

**Table 5:** Robustness test results 2.

Variables	(1)	(2)	(3)	(4)	(5)
Policy	0.1083*** (0.0310)	0.0942*** (0.0258)	0.0993*** (0.0247)	0.0783*** (0.0216)	0.0694*** (0.0157)
Smart city	0.0012 (0.0040)				0.0098** (0.0046)
Low carbon city		0.0442*** (0.0043)			0.0327*** (0.0045)
Broadband China			0.0204*** (0.0045)		0.0064* (0.0043)
Innovative city				0.0881*** (0.0052)	0.0820*** (0.0051)
Control variable linear term	Yes	Yes	Yes	Yes	Yes
Control variable quadratic term	Yes	Yes	Yes	Yes	Yes
City FE	Yes	Yes	Yes	Yes	Yes
Year FE	Yes	Yes	Yes	Yes	Yes
N	4496	4496	4496	4496	4496

***, **, and * indicates statistical significance at 1%, 5%, and 10% levels, respectively. Robust standard errors are in parentheses.

#### Reset double ML model

To avoid the impact of the double ML model imparting bias on the conclusions, we conduct robustness tests by varying the sample splitting ratio, the ML algorithm, and the model estimation form. First, we change the sample split ratio of the double ML model from 1:4 to 3:7 and 1:3. Second, we replace the Lasso ML algorithm with random forest (RF), gradient boosting (GBT), and BP neural network (BNN). Third, we replace the partial linear model based on the dual ML with a more generalized interactive model, using the following main and auxiliary regressions for the analysis:

8 $$ Y_{it} = g(Policy_{it} ,X_{it} ) + U_{it} $$

9 $$ Policy_{it} = m(X_{it} ) + V_{it} $$

among them, the meanings of each variable are the same as Eqs. (1) and (2).

The estimated coefficients for the treatment effects are obtained from the interactive model as follows:

10 $$ \tilde{\theta }_{1} = E\left[ {g(Event_{it} = 1,X_{it} )} \right] - E\left[ {g(Event_{it} = 0,X_{it} )} \right] $$

Table 6 presents the regression results after resetting the double ML model, revealing that the sample split ratio, ML algorithm, and the model estimation form in double ML model did not affect the conclusion that the Made in China 2025 strategy promotes urban green economic growth, and only alters the magnitude of the policy effect, once again validating the robustness of our conclusions.

**Table 6:** Robustness test results 3.

Variables	(1)	(2)	(3)	(4)	(5)	(6)
	3:7	1:3	RF	GBT	BNN	Interactive model
Policy	0.1381*** (0.0319)	0.1462*** (0.0370)	0.1763*** (0.0328)	0.1235*** (0.0356)	0.1194*** (0.0347)	0.1325*** (0.0294)
Control variable linear term	Yes	Yes	Yes	Yes	Yes	Yes
Control variable quadratic term	Yes	Yes	Yes	Yes	Yes	Yes
City FE	Yes	Yes	Yes	Yes	Yes	Yes
Year FE	Yes	Yes	Yes	Yes	Yes	Yes
N	4496	4496	4496	4496	4496	4496

***, **, and * indicates statistical significance at 1%, 5%, and 10% levels, respectively. Robust standard errors are in parentheses.

#### Difference-in-differences model

To further verify the robustness of the estimation results, we use traditional econometric models for regression. Based on the difference-in-differences (DID) model, a synthetic difference-in-differences (SDID) model is constructed by combining the synthetic control method^68^. It constructs a composite control group with a similar pre-trend to the treatment group by linearly combining several individuals in the control group, and compares it with the treatment group^69^. Table 7 presents the regression results of traditional DID model and SDID model. The estimated coefficient of the Made in China 2025 policy remains significantly positive at the 1% level, which once again verifies the robustness of the study’s findings.

**Table 7:** Robustness test results 4.

Variables	(1)	(2)
	Traditional DID	SDID
Policy	0.0937*** (0.0284)	0.1095*** (0.0301)
Control variable linear term	Yes	Yes
City FE	Yes	Yes
Year FE	Yes	Yes
N	4496	4496

***, **, and * indicate statistical significance at 1%, 5%, and 10% levels, respectively. Robust standard errors are in parentheses.

### Mechanism verification

This section conducts mechanism verification from four perspectives of green technology advancement, energy consumption structure, industrial structure, and environmental supervision. The positive impacts of the Made in China 2025 strategy on green technology advancement, energy consumption structure optimization, industrial structure upgrading, and strengthening environmental supervision are empirically examined using a dual ML model (see Table A.1 in the Online Appendix for details). Referencing Farbmacher et al. (2022)^39^ for causal mediating effect analysis of double ML (see the Appendix for details), we test the transmission mechanism of the Made in China 2025 strategy on green economic growth based on the Lasso algorithm, presenting the results in Table 8. The findings show that the total effects under different mediating paths are all significantly positive at the 1% level, verifying that the Made in China 2025 strategy positively promotes urban green economic growth.

**Table 8:** Mechanism test results.

Variables	Total effect	Direct effect		Indirect effect	
		Treatment group	Control group	Treatment group	Control group
Gtech	0.2908*** (0.0523)	0.1863** (0.0605)	0.1193*** (0.0306)	0.1715** (0.0653)	0.1049*** (0.0347)
Energy	0.2878*** (0.0421)	0.1773** (0.0710)	0.1268*** (0.0401)	0.1610** (0.0597)	0.1105*** (0.0231)
Indus	0.3365*** (0.0592)	0.2386*** (0.0405)	0.1772** (0.0619)	0.1593** (0.0415)	0.0979** (0.0378)
Superv	0.3796*** (0.0632)	0.2150** (0.0632)	0.1964*** (0.0310)	0.1832*** (0.0421)	0.1646*** (0.0534)

***, **, and * indicates statistical significance at 1%, 5%, and 10% levels, respectively. Robust standard errors are in parentheses.

#### Mechanism of green technology advancement

The indirect effect of green technological innovation is significantly positive for both the treatment and control groups. After stripping out the path of green technology advancement, the direct effects of the treatment and control groups remain significantly positive, indicating that the increase in the level of green technological innovation brought about by the Made in China 2025 strategy significantly promotes urban green economic growth. The Made in China 2025 strategy proposes to strengthen financial and tax policy support, intellectual property protection, and talent training systems. Through the implementation of policy incentives, pilot cities have fostered the concentration of high-technology enterprises and scientific and technological talent cultivation, exerting a knowledge spillover effect that further promotes green technology advancement. At the same time, policy preferences have stimulated the demand for innovation in energy conservation and emissions reduction, which raises enterprises’ motivation to engage in green innovation activities. Green technology advancement helps cities achieve an intensive development model, bringing multiple dividends such as lower resource consumption, reduced pollution emissions, and improved production efficiency, which subsequently promotes green economic growth.

#### Mechanism of energy consumption structure

The indirect effect of energy consumption structure is significantly positive for the treatment and control groups, while the direct effect of the Made in China 2025 pilot policy on green economic growth remains significantly positive, indicating that the policy promotes urban green economic growth through energy consumption structure optimization. The policy encourages the introduction of clean energy into production processes, reducing pressure on enterprise performance and the cost of clean energy use, which helps enterprises to reduce traditional energy consumption that is dominated by coal and optimize the energy structure to promote green urban development.

#### Mechanism of industrial structure

The indirect effects of industrial structure on the treatment and control groups are significantly positive. After stripping out the path of industrial structure upgrading, the direct effects remain significantly positive for both groups, indicating that the Made in China 2025 strategy promotes urban green economic growth through industrial structure optimization. Deepening the restructuring of the manufacturing industry is a strategic task specified in Made in China 2025. Pilot cities focus on transforming and guiding the traditional manufacturing industry toward high-end, intelligent equipment upgrades and digital transformation, driving the regional industrial structure toward rationalization and advancement to achieve rational allocation of resources. Upgrading industrial structure is a prerequisite for cities to advance intensive growth and sustainable development. By assuming the roles of “resource converter” and “pollutant controller,” industrial upgrading can continue to release the dividends of industrial structure, optimize resource allocation, and improve production efficiency, establishing strong support for green economic growth.

#### Mechanism of environmental supervision

The treatment and control groups of environmental supervision has a positive indirect effect in the process of the Made in China 2025 pilot policy affecting green economic growth that is significant at the 1% level, affirming the transmission path of environmental supervision. The Made in China 2025 strategy states that energy consumption, material consumption, and pollutant emissions per unit of industrial added value in key industries should reach the world’s advanced level by 2025. This requires pilot cities to consolidate and propagate the effectiveness of green development by strengthening environmental supervision while promoting the manufacturing sector’s green development. Strengthening environmental supervision promotes enterprises’ energy saving and emissions reduction through innovative compensation effects, while restraining enterprises’ emissions behaviors by tightening environmental protection policies, promoting environmental legislation, and increasing penalties to advance green urban development. Based on the above analysis, Hypothesis 2 is validated.

### Heterogeneity analysis

#### Heterogeneity of manufacturing agglomeration

To reduce production and transaction costs and realize economies of scale and scope, the manufacturing industry tends to accelerate its growth through agglomeration, exerting an “oasis effect”^70^. Cities with a high degree of manufacturing agglomeration are prone to scale and knowledge spillover effects, which amplify the agglomeration functions of talent, capital, and technology, strengthening the effectiveness of pilot policies. Based on this, we use the locational entropy of manufacturing employees to measure the degree of urban manufacturing agglomeration in the year (2015) before policy implementation, using the median to divide the full sample of cities into high and low agglomeration groups. Columns (1) and (2) in Table 9 reveal that the Made in China 2025 pilot policy has a stronger effect in promoting green economic growth in cities with high manufacturing concentration compared to those with low concentration. The rationale for this outcome may be that cities with a high concentration of manufacturing industries has large population and developed economy, which is conducive to leveraging agglomeration economies and knowledge spillover effects. Meanwhile, they are able to offer greater policy concessions by virtue of economic scale, public services, infrastructure, and other advantages. These benefits can attract the clustering of productive services and the influx of innovative elements such as R&D talent, accelerating the transformation and upgrading of the manufacturing industry and the integration and advancement of green technologies, empowering the green urban development.

**Table 9:** Heterogeneity analysis results.

Variables	(1)	(2)	(3)	(4)	(5)	(6)
	Manufacturing agglomeration		Industrial intelligence		Digital finance	
	High	Low	High	Low	High	Low
Policy	0.2048*** (0.0505)	0.0541* (0.0389)	0.1732*** (0.0409)	0.0824** (0.0381)	0.1494*** (0.0358)	0.0765** (0.0290)
Control variable linear term	Yes	Yes	Yes	Yes	Yes	Yes
Control variable quadratic term	Yes	Yes	Yes	Yes	Yes	Yes
City FE	Yes	Yes	Yes	Yes	Yes	Yes
Year FE	Yes	Yes	Yes	Yes	Yes	Yes
N	2248	2248	2248	2248	2248	2248

***, **, and * indicates statistical significance at 1%, 5%, and 10% levels, respectively. Robust standard errors are in parentheses.

#### Heterogeneity of industrial intelligence

As a landmark technology for the integration of the new scientific and technological revolution with manufacturing, industrial intelligence is a new approach for advancing the green transformation of manufacturing production methods. Based on this, we use the density of industrial robot installations to measure the level of industrial intelligence in cities in the year (2015) prior to policy implementation^71^, using the median to classify the full sample of cities into high and low level groups. Columns (3) and (4) in Table 9 reveals that the Made in China 2025 pilot policy has a stronger driving effect on the green economic growth of highly industrial intelligent cities. The rationale for this outcome may be that with the accumulation of smart factories, technologies, and equipment, a high degree of industrial intelligence is more likely to leverage the green development effects of pilot policies. For cities where the development of industrial intelligence is in its infancy or has not yet begun, the cost of information and knowledge required for enterprises to undertake technological R&D is higher, reducing the motivation and incentive to conduct innovative activities, diminishing the pilot policy’s contribution to green economic growth.

#### Heterogeneity of digital finance

As a fusion of traditional finance and information technology, digital finance has a positive impact on the development of the manufacturing industry by virtue of its advantages of low financing thresholds, fast mobile payments, and wide range of services^72^. Cities with a high degree of digital finance development have abundant financial resources and well-developed financial infrastructure that provide enterprises with more complete financial services, with subsequent influence on the effects of pilot policies. We use the Peking University Digital Inclusive Finance Index to measure the level of digital financial development in cities in the year (2015) prior to policy implementation, using the median to divide the full sample of cities into high and low level groups. Columns (5) and (6) in Table 9 reveal that the Made in China 2025 pilot policy has a stronger driving effect on the green economic growth of cities with highly developed digital finance. The rationale for this outcome may be that cities with a high degree of digital finance development can fully leverage the universality of financial resources, provide financial supply for environmentally friendly and technology-intensive enterprises, effectively alleviate the mismatch of financial capital supply, and provide financial security for enterprises to conduct green technology R&D. Digital finance also makes enterprises’ information more transparent through a rich array of data access channels, which strengthens government pollution regulation and public environmental supervision and compels enterprises to engage in green technological innovation to promote green economic growth.

## Conclusion and policy recommendation

### Conclusions

This study examines the impact of the Made in China 2025 strategy on urban green economic growth using the double ML model based on panel data for 281 Chinese cities from 2006 to 2021. The relevant research results are threefold. First, the Made in China 2025 strategy significantly promotes urban green economic growth; a conclusion that is supported by a series of robustness tests. Second, regarding mechanisms, the Made in China 2025 strategy promotes urban green economic growth through green technology advancement, energy consumption structure optimization, industrial structure upgrading, and strengthening of environmental supervision. Third, the heterogeneity analysis reveals that the Made in China 2025 strategy has a stronger driving effect on green economic growth for cities with a high concentration of manufacturing and high degrees of industrial intelligence and digital finance.

### policy recommendations

We next propose specific policy recommendations based on our findings. First, policymakers should summarize the experience of building pilot cities and create a strategic model to advance the transformation and upgrading of the manufacturing industry to drive green urban development. The Made in China 2025 pilot policy effectively promotes green economic growth and highlights the significance of the transformation and upgrading of the manufacturing industry to empower sustainable urban development. The government should strengthen the model and publicize summaries of successful cases of manufacturing development in pilot cities to promote the experience of manufacturing transformation and upgrading by producing typical samples to guide the transformation of the manufacturing industry to intelligence and greening. Policies should endeavor to optimize the industrial structure and production system of the manufacturing industry to create a solid real economy support for high-quality urban development.

Second, policymakers should explore the multidimensional driving paths of urban green economic growth and actively stimulate the green development dividend of pilot policies by increasing support for enterprise-specific technologies, subsidizing R&D in areas of energy conservation and emissions reduction, consumption reduction and efficiency, recycling and pollution prevention, and promoting the progress of green technologies. The elimination of outdated production capacity must be accelerated and the low-carbon transformation of traditional industries must be targeted, while guiding the clustering of high-tech industries, optimizing cities’ industrial structure, and driving industrial structure upgrading. Policymakers can regulate enterprises’ production practices and enhance the effectiveness of environmental supervision by improving the system of environmental information disclosure and mechanisms of rewards and penalties for pollution discharge. In addition, strategies should consider cities’ own resource endowment, promote large-scale production of new energy, encourage enterprises to increase the proportion of clean energy use, and optimize the structure of energy consumption.

Third, policymakers should engage a combination of urban development characteristics and strategic policy implementation to empower green urban development, actively promoting optimization of manufacturing industry structure, and accelerating the development of high-technology industries under the guidance of policies and the market to promote high-quality development and agglomeration of the manufacturing industry. At the same time, the government should strive to popularize the industrial internet, promote the construction of smart factories and the application of smart equipment, increase investment in R&D to advance industrial intelligence, and actively cultivate new modes and forms of industrial intelligence. In addition, new infrastructure construction must be accelerated, the application of information technology must be strengthened, and digital financial services must be deepened to ease the financing constraints for enterprises conducting R&D on green technologies and to help cities develop in a high-quality manner.

### Supplementary Information

Supplementary Information.

## Data Availability

The datasets used or analysed during the current study are available from the corresponding author on reasonable request.
