# Supplementary material for: A double machine learning model for measuring the impact of the Made in China 2025 strategy on green economic growth
Source: Sci Rep. 2024 May 26;14:12026. doi: 10.1038/s41598-024-62916-0 (PMC11128443; doi:10.1038/s41598-024-62916-0)
Supplement: Supplementary file 1 — Supplementary Information. [file 41598_2024_62916_MOESM1_ESM.docx]

**Supplementary Material**

**Appendix**

We examine the effect of the Made in China 2025 strategy on the proposed mechanism variables based on the double ML model. The results in Table A.1 demonstrate that the coefficient of the Made in China 2025 pilot policy is significantly positive, indicating that the policy promotes green technology advancement, energy consumption structure optimization, industrial structure upgrading, and strengthening of environmental supervision.

Table A.1. Regression results of Made in China 2025 strategy on mechanism variables

| Variables | (1) | (2) | (3) | (4) |
| --- | --- | --- | --- | --- |
|  | Gtech | Energy | Indus | Superv |
| Policy | 0.0738***  (0.0152) | 0.1263***  (0. 0265) | 0.0574**  (0.0240) | 0.2048***  (0.0381) |
| Control variable linear term | YES | YES | YES | YES |
| Control variable quadratic term | YES | YES | YES | YES |
| City FE | YES | YES | YES | YES |
| Year FE | YES | YES | YES | YES |
| N | 4496 | 4496 | 4496 | 4496 |

Note: ***, **, and * indicates statistical significance at 1%, 5%, and 10% levels, respectively. Robust standard errors are in parentheses.

In contrast to traditional mediating effects analyses, causal mediating effects analysis assumes that the mediator (*M*) depends on treatment status (*d*) and that the explained variable (*Y*) depends on both *M* and *d*. Hence, the direct effect (*θ*) is determined by the individual mediator state *M*(*d*) based on the change in treatment state, and the indirect effect (*δ*) is determined by *d* based on the change in mediator state. This suggests that the mediator *M* and *d* are separate from each other (counterfactual framework), which can be expressed separately as follows:

$\begin{matrix} \theta(1)=E[Y(1,M(1))-Y(0,M(1))] \\ \theta(0)=E[Y(1,M(0))-Y(0,M(0))] \\ \theta(1)=E[Y(1,M(1))-Y(1,M(0))] \\ \theta(0)=E[Y(0,M(1))-Y(0,M(0))] \end{matrix}$ (A.1)

Interpretation of causal mediating effects analysis also differs, in addition to the representation of direct and indirect effects diverging from traditional mediating effects analysis. This study tests the mechanistic pathways of mediators based on causal mediating effects analysis, using the indirect effect as an example for unique illustration. The indirect effects of the treatment and control groups in the study are not specific to pilot or nonpilot cities of Made in China 2025, but can be further interpreted as changes in the explained variables generated by the change in mediating factors from the untreated state to the treated state in the initial circumstance when a city receives the treatment or does not receive the treatment. As the initial treatment status varies across individual cities, indirect effects should also vary. We made this clarification to avoid any reader confusion about the narrative in the text.
